# Round the Hospitals

**Published:** 1921-07-30

**Authors:** 


### ROUND THE HOSPITALS.

Members of the nursing profession who desire to become registered nurses are now invited to take the first step in this direction by applying for an Application Form for Registration to the Registrar of the General Nursing Council, 12 York Gate, Regent's Park. Applications for these forms should state to which part or parts of the Register the applicant desires to be admitted; *i.e.* (1) General; (2) Male; (3) Mental; (4) Sick Children; (5) Fever; the form for each part is different, and this course will ensure that they get the particular form which applies to their case. A long envelope, stamped, should be enclosed with each application for a form, or forms. Copies of the Rules may also now be obtained from the Registrar, price 1s., or by post 1s. 2d., and we advise all nurses to secure one. These Rules regulate the conditions of admission to the Register of Existing Nurses (*i.e.* those in practice before November 1919), and Intermediate Nurses (*i.e.* those who had not completed their training by November 1, 1919), deal with the formation, maintenance, and publication of the Register, and prescribe the causes for which and the conditions under which nurses may be removed from the Register, and the procedure for the restoration of such names. Rules with respect to the uniform or badge which may be worn by registered nurses are not at present included, but will be published in due course.

---

In our issue of June 25, page 222, we published the regulations for the diploma in nursing of the Leeds University which have been adopted by the Council of the University and sanctioned by the Court. Candidates for examination for the diploma must have completed four years' training in a general hospital recognised by the University of Leeds; they must furnish evidence of having attained

**ROUND THE HOSPITALS**—*(continued)*.

an adequate standard in general education satisfactory to the University, and they will be required to attend courses of lectures in the University of Leeds on social economics or other approved subjects. The examinations for the diploma will take place at a suitable time to allow the results to be published at the same time as the results of the other University examinations, namely, in December and June. A small committee to assess the general education of the candidates presenting themselves has now been appointed by the University Senate. Its members are the Vice-Chancellor of the University (Sir Michael Sadler), the Dean of the Faculty of Medicine (Professor J. Kay Jamieson), Professor C. M. Gillespie, Dr. Maxwell Telling, and the Lady Superintendent of the Leeds General Infirmary.

A conference was held on July 19 between a deputation from the British Hospitals Association and the Salaries Committee of the College of Nursing. We trust the good effects of this will shortly become apparent in an agreed and standardised scale of payment for all grades of nurses and by all institutions. Much has been done recently, but there is room for considerable improvement in many directions, and the best way to get matters of this kind settled is by round-table conferences between representatives of each side.

Despite apparently authoritative information to the contrary we are informed that the College of Nursing and Cowdray Sister-Tutor Scholarships were not awarded until Thursday, July 21; seeing that some of the candidates were only interviewed by the Studentship Committee the previous day, it will be agreed that no time has been lost in deciding on the awards which we are now able to publish. The successful candidates for the College Scholarships are Miss Doris Taylor, who was trained at St. Bartholomew's Hospital, Rochester, and the National Hospital, Queen Square; and Miss Bertha M. B. Haughton, trained at the Middlesex Hospital. The two Cowdray Scholarships, for candidates trained in Aberdeenshire or Sussex, are awarded to Miss Margery Poole, trained at the Royal Sussex County Hospital, Brighton, and Miss Faith Moulson, trained at the East Sussex Hospital, Hastings; while the London Centre Scholarship has been awarded to Miss Olive Margaret Billinghurst, trained at the London Hospital. We congratulate the successful candidates and hope they will do useful work on the termination of their course at King's College for Women.

Our columns show weekly that the demand for sister-tutors is growing; there is no doubt it will continue to grow, and those nurses who are privileged to take the King's College course have the best of prospects of interesting and remunerative posts. The Great Northern Central Hospital, which has 207 occupied beds, is a Metropolitan hospital which has just decided to appoint a sister-tutor in order to maintain its training at the highest standard. Its staff is increasing and the committee desires that more individual training shall be given. Two of the nurses of this hospital, Nurses Gregory and Williamson, have been awarded the silver and bronze medals respectively, as they passed the senior examination with distinction and merited special recognition.

Inspection is not a particularly popular mission in life, and those who were inspected used to experience both suspicion and dislike to those appointed to perform that office for their benefit. The inspectors attached to Queen Victoria's Jubilee Institute have changed all that. Their visits are anticipated with pleasure by the nurses within their beat, often lonely women with no experienced fellow-nurse to consult with, and their influence has become fruitful for good in a hundred practical directions. At their recent meeting the Council of Queen Victoria's Jubilee Institute put it on record by special resolution that "it is largely due to the faithful and intelligent service of the inspectors that the position of the Queen's nurses throughout the United Kingdom stands where it does at present." The financial difficulties of the Institute are still unsolved, though the treasurer can now see his way to the end of the year. The national scheme for nursing insured persons will, it is hoped, come into operation shortly. If it eases the situation, as it ought to do very materially, for the local associations, they should have more to spare in support of the central organisation. The Lansdowne House Ball has brought in over £3,000.

A special meeting of the Board of Managers was held on July 13 at the Swansea General Hospital, at which deputations from the Carmarthen, Llanelly, and Port Talbot Hospitals attended. The purpose was to protest against the exclusion of nurses trained in small hospitals from the College of Nursing, and from participation in the Nation's Fund for Nurses. The resolution before the meeting was couched in very bitter language, and was coupled with another to the effect that the Swansea Hospital "severs its connection with and withdraws its support from the College of Nursing." Neither resolution found favour with the meeting, and, after a discussion lasting nearly two hours, an amendment, moved by the Rev. H. S. Mander, was substituted, in which, after expressing general approval of the proposals of the College for the future training and standardisation of the nursing profession, the Board states that the present conditions of membership inflict a grave hardship upon a large number of nurses trained during the last twenty years, and calls upon the College to modify its rules retrospectively, so as to admit to membership all hospital-trained and certificated nurses.

The Marchioness of Salisbury was able to announce at the recent annual meeting of the Hertfordshire County Nursing Association that they had raised over £2,000 more throughout the county in annual subscriptions in 1920 than in the previous year. The entire cost came to £15,045, and of this voluntary subscriptions amounted to £9,561. This

**ROUND THE HOSPITALS**—(*continued*).

is a most encouraging sign of the times, and shows what can be done under a zealous central organisation which understands its business.

On the occasion of the second prize-giving at the St. Marylebone Infirmary, on July 14, the probationers were addressed by Sir Arthur Newsholme, K.C.B., late Principal Medical Officer of the Local Government Board. He put forward the suggestion, which has seen the light of day before, that now that the nursing profession is more highly educated it might be useful to employ a superior kind of wardmaid, as in America, to work under the trained nurse, who in time would become less a nurse for those who were sick than a health visitor to prevent disease. In the same way, he hopes the function of the general practitioner will in time to come be to keep people from being ill rather than to treat them when sick. He congratulated the Marylebone Guardians upon the pioneer step they have taken by asking consultants to visit the infirmary. An enjoyable afternoon was completed with tea served in the big garden, where the nurses have a grass and an asphalt tennis court.

The deepest regret is felt by all connected with the Royal Portsmouth Hospital at the retirement of Miss Alcock, R.R.C., who has been matron for ten years, and that regret was given verbal and material expression to in the presentations made to her last week. Both Sir Harold Pink, who was in the chair, and Mr. T. H. F. Lapthorn spoke in the highest terms of Miss Alcock, whose record at the hospital, said the latter, might be summarised in the one word "success." Not only in the nursing but in the administrative side of the matron's department has Miss Alcock left a worthy heritage to her successor; during her period of office the staff has been increased, its hours reduced, a sister-tutor appointed, and the Hospital Ladies' Linen League, which Miss Alcock initiated, has become a thriving institution, from which £250 yearly is derived. The presentations were a cheque for £75 18s. from members of the management committee, the Governors of the hospital, and the outside public; a couch from past and present nurses of the hospital; and a cushion, with hand-painted cover, from the domestic staff, whose efficient service in the past Miss Alcock particularly referred to.

The nursing-home venture seems to be attracting a good many matrons at present. One of the latest to embark on this work is Miss Constance Crookenden, R.R.C., who after eight years' service as matron of Addenbrooke's Hospital has resigned in order to become proprietor of a nursing home at Brighton. Miss Crookenden's nursing and administrative experience—she was Principal Matron of the 1st Eastern General Hospital, Cambridge, during the war, which contained 1,750 beds—should eminently fit her for such a post. She was trained at St. Thomas's Hospital, and after private nursing for one year she was appointed matron of the Cray Valley Hospital. Here she remained for five and a-half years, when she was unanimously elected matron of Addenbrooke's Hospital on the resignation of Miss Montgomery. Miss Crookenden's successor at Cambridge will be Miss Forster-Feather, also a "Nightingale" nurse, who has been appointed out of thirty-one candidates. On completion of her training she did one year's military work in France, and has since held the post of sister at the Herefordshire General Hospital, at the Derbyshire Royal Infirmary, and night sister at Queen Mary's Hospital for the East End. She then went to Charing Cross Hospital as pupil housekeeper, and subsequently was successively home sister, assistant matron, and matron of the Royal Gwent Hospital, Newport, Monmouthshire. Miss Forster-Feather will take up her duties at Cambridge in September.

The South African Trained Nurses' Association is taking up the case of nurses requiring treatment in hospitals. The present ruling at the Transvaal and at the Cape is that a nurse receives free attendance in the hospital in which she works but in no other, while private nurses are not entitled to it. The result is that a nurse must either pay a sum she cannot afford or be treated as a pauper. We agree with the editor of the *S.A.N. Record* that the principle should be established which accords free hospital treatment both to nurse and doctor when required, and that this would lead to a more brotherly feeling between doctors, nurses and hospitals.

The distribution of grants from the Tribute Fund has stirred up the vexed question, "What is a trained nurse?" We trust shortly to have an authoritative answer in the General Nursing Council's definition of the existing nurse as eligible for registration. But meantime there is very little practical difficulty in ascertaining whether an applicant for assistance is a genuine nurse or merely a woman who has on occasions acted as attendant to an invalid. The Tribute Fund has the co-operation of experienced nurses in allocating help to members of the profession, and numerous as are the shades of distinction among the trained, there is no doubt that a real dividing-line separates the trained from the untrained.

The East Ham Education Committee has excited comment by advertising for a school nurse who must not be over thirty. No slur, however, has been cast on older members of the profession by the desire expressed for a comparatively young nurse to fill the post. The work of school nursing requires a certain apprenticeship, and is better undertaken soon after leaving the training-school, when the powers of receptivity and adaptation are still elastic. School-nursing posts tend to become permanent, and there is no suggestion that the successful applicant at East Ham will be deprived of her appointment after she reaches the age of thirty.

